# Use of Brief Messages Based on Behavior Change Techniques to Encourage Medication Adherence in People With Type 2 Diabetes: Developmental Studies

**DOI:** 10.2196/15989

**Published:** 2020-05-13

**Authors:** Yvonne Kiera Bartlett, Andrew Farmer, Rustam Rea, David P French

**Affiliations:** 1 Manchester Centre for Health Psychology University of Manchester Manchester United Kingdom; 2 Nuffield Department of Primary Care Health Sciences University of Oxford Oxford United Kingdom; 3 Oxford Centre for Diabetes, Endocrinology and Metabolism Oxford University Hospitals Foundation Trust Oxford United Kingdom; 4 National Institute for Health Research Oxford Biomedical Research Centre Oxford University Hospitals National Health Service Foundation Trust Oxford United Kingdom

**Keywords:** behavior change, behavior change techniques, type 2 diabetes, brief messages, mHealth, medication adherence

## Abstract

**Background:**

Brief messages are a promising way to improve adherence to medication for people with type 2 diabetes. However, it is often unclear how messages have been developed and their precise content, making it difficult to ascertain why certain messages are successful and some are not.

**Objective:**

The goal of the research was to develop messages that have proven fidelity to specified evidence-derived behavior change techniques (BCTs) and are acceptable to people with type 2 diabetes.

**Methods:**

Four studies were conducted: (1) a workshop (n=21) where behavioral change researchers and health care professionals developed messages based on specific BCTs or beliefs or concerns related to taking medication, (2) a focus group study with people with type 2 diabetes (n=23) to assess acceptability of the approach, (3) a survey to ascertain the acceptability of a subset of messages to people with type 2 diabetes (n=61) and, (4) a survey with behavior change researchers to assess the fidelity of a subset of messages to their intended BCT (n=18).

**Results:**

In study 1, 371 messages based on 38 BCTs and beliefs/concerns were developed. Workshop participants rated BCTs to be relevant to medication adherence (mean 7.12/10 [SD 1.55]) and messages to have good fidelity (mean 7.42/10 [SD 1.19]). In study 2, the approach of providing medication adherence support through text messages was found to be acceptable. In study 3, mean acceptability of all BCTs was found to be above the midpoint (mean 3.49/5 [SD 0.26]). In study 4, mean fidelity for all BCTs was found to be above the midpoint (mean 7.61/10 [SD 1.38]).

**Conclusions:**

A library of brief messages acceptable to people with type 2 diabetes and representative of specific evidence-derived BCTs was developed. This approach allowed brief messages to be developed with known content that can be used to test theory.

## Introduction

Type 2 diabetes leads to high levels of glucose in the blood; if left uncontrolled, the condition can lead to a wide range of micro- and macrovascular complications including problems with the heart, feet, and eyesight [1]. There are effective tablet medications available that can help control blood glucose and reduce the risk of complications in people with type 2 diabetes. However, adherence to and persistence with tablet medication is poor for at least half the population of people with type 2 diabetes prescribed these medications [2]. If less than 80% of the prescribed medication is taken, only half the expected reduction in blood glucose control is seen, increasing the risk of complications for this population [3].

A recent Cochrane review concluded that medication adherence interventions have led to only modest increases in adherence, and studies with the lowest risk of bias had the lowest efficacy [4]. The authors proposed that novel approaches to this problem are needed [4]. One approach that has shown promise to improve medication adherence in people with type 2 diabetes is the delivery of brief messages. In a review of 15 interventions, there was some evidence of effectiveness, but there were weaknesses in the study designs [5]. Further, the review authors commented that the interventions were not well described, and the majority did not report being based on any explicit theoretical framework [5]. It has been noted that for brief message interventions “the content is the central driver of the behavior change” [6]; it is therefore essential that the content is well described so that whether a change in behavior does or does not occur it can be explained, and the intervention can be improved as needed. However, in many published reports, in addition to the content of messages being unclear, the development process can also be unclear with little reported about exactly how information from formative work is incorporated into the final intervention. This lack of transparency has led to the development process being described as a black box [7].

There are multiple ways to develop interventions to change behavior, and different approaches may be suitable for different contexts [8]. These approaches vary in the emphasis they place on the extent to which interventions should be based on theory and evidence of effectiveness and whether they should be developed and/or extensively tested with the intended target population. More specifically in the electronic health area, there are a growing number of approaches that focus on developing technology-based interventions (eg, the person-centered approach [9], behavioral intervention technology model [10], intervention mapping combined with the behavioral intervention technology model [11], and the mobile health development and evaluation framework [12]). However, these models provide a broad overview for development of many different types of technology-based interventions, which are often complex with multiple components. For this reason, the actual process of writing content such as brief messages is not often explained in detail. In cases where the actual process of writing the messages is reported or is the focus of the suggested procedure [13], it seems that it is conducted by a small number of people in the intervention team. Having a small team of people involved in the intervention drafting the messages may lead to a similarity in the approaches used compared with what has gone before rather than exploring novel approaches to the problem of medication adherence.

This research developed and refined message content using specific behavior change techniques (BCTs) to develop messages or as a means to code messages for content. BCTs are described as the active ingredients of an intervention [14]. They are the irreducible components of the intervention such as goal-setting or self-monitoring of behavior. Thus far, 93 such BCTs have been identified and defined in a taxonomy [15]. In cases where the BCT content of brief messages related to diabetes self-management have been defined in published reports, thus far relatively few of the 93 BCTs identified in the v1 taxonomy [15] have been used. In two reviews in the area, one identified 8 BCTs across 7 studies [16] and the other 16 BCTs across 6 studies [17].

The studies described in this paper form part of a larger body of work to develop a brief message service that could be used by people with type 2 diabetes within the UK National Health Service (NHS). Given the lack of effectiveness of existing interventions to promote medication adherence [4], it was important to identify as many potentially effective BCTs that could be delivered by brief message as possible. Prior to the work reported here, a rapid systematic review of systematic reviews was conducted [18]. This review highlighted a wide range of BCTs that existing quantitative systematic reviews had previously identified as being associated with changes in medication adherence across a range of chronic physical health conditions. In addition, qualitative reviews were examined to identify specific beliefs and concerns associated with taking medication for people with type 2 diabetes to ensure that novel insights inductively obtained from people with type 2 diabetes regarding their medication adherence were included [18]. Of the 46 BCTs identified, 13 were either not considered suitable for delivery via brief messages or, in delivering through brief messages, the content could be covered by another BCT (for excluded BCTs and reasons see Multimedia Appendix 1, Table A), 2 of the BCTs were considered more appropriately represented by 3 of the beliefs and concerns (see Table A for details).

This paper presents a transparent process of brief message development that aimed to ensure the message content had high fidelity to the intended BCTs. Further, the inclusion of relevant stakeholders at all stages of the research was deemed essential to ensure that the messages were acceptable to the target population. People with type 2 diabetes were included both as participants in the research studies but also in Patient and Public Involvement (PPI) panels. Members of the PPI panel participated in key meetings and research decisions, as well as reviewing protocols and participant-facing materials prior to ethical approval.

**Figure 1:**
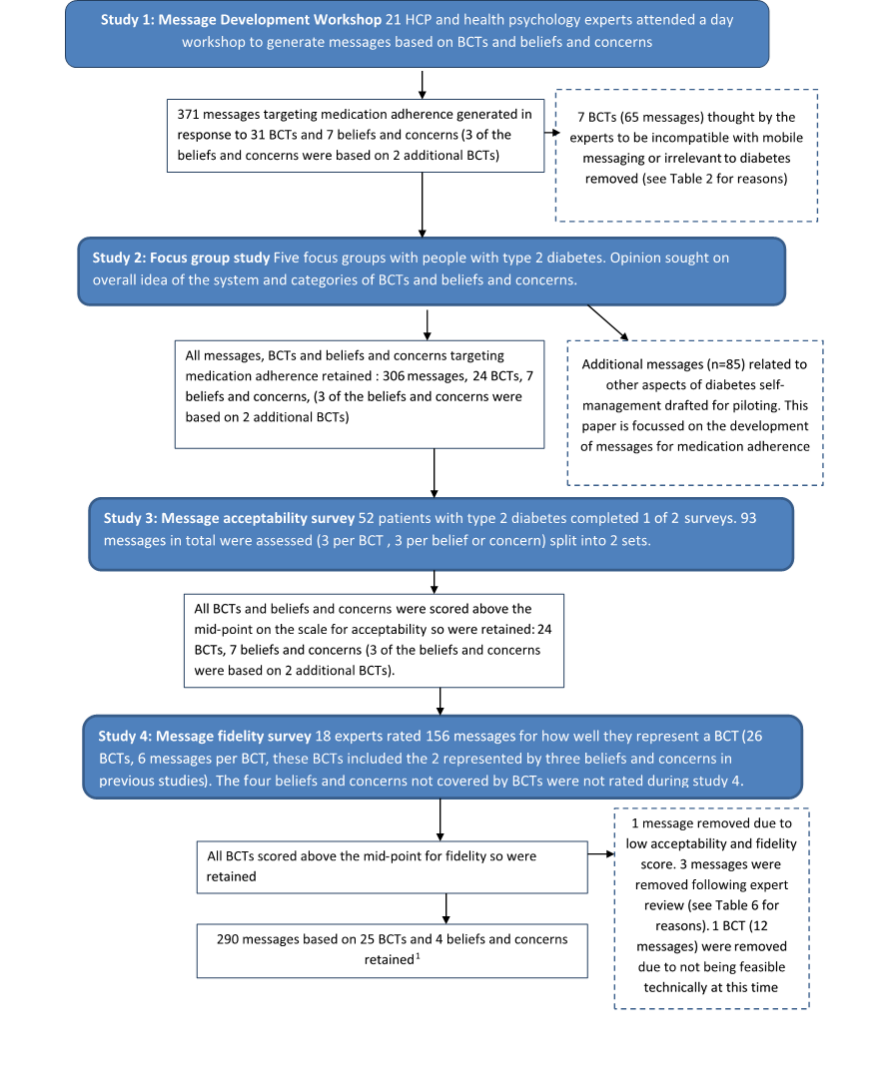
Development of messages targeting medication adherence through the four studies. BCT: behavior change technique; ^1^Three of the beliefs and concerns are represented by 2 additional BCTs here.

This paper reports the process of message development through 4 studies and decisions taken following each study. The purpose of this research was to develop a library of messages that are acceptable and represent explicit BCTs, identify any BCTs that were not suitable for delivery through brief messages, and identify any messages or BCTs that should be removed prior to further development. The studies reported on are (1) a workshop to develop a library of messages based on specified BCTs and beliefs and concerns, (2) a focus group study to look at acceptability of the concept, (3) an online survey to assess the acceptability of the BCTs and messages developed during study 1 to people with type 2 diabetes, and (4) an online survey to assess the acceptable messages from study 3 for fidelity to the intended BCTs (see Figure 1 for an overview of message development through the four studies).

## Methods

### Ethical Approval

Ethical approval for the studies 1 and 4 was granted by the University of Oxford Central University Research Ethics Committee Medical Sciences Interdivisional Research Ethics Committee (ref: R50752/RE001). Ethical approval for studies 2 and 3 was granted by the NHS North West–Greater Manchester West Research Ethics Committee (ref: 17/NW/0224).

### Study 1: Message Development Workshop

#### Aim

The aim of the workshop was to develop a library of messages based on specified BCTs and beliefs and concerns.

#### Recruitment

Participants were expert researchers in the fields of behavior change or medication adherence or health care professionals (HCPs) involved in diabetes care. Potential participants were identified from the research team’s knowledge of those active in these fields in the United Kingdom or Europe. A total of 31 invitations were sent by email, with a single reminder email.

#### Procedure

Participants were invited to attend a 1-day workshop in Manchester, England. An information sheet was sent to participants ahead of time and informed consent was taken on the day of the workshop. The day was split into 4 sessions. For the first 3 sessions, behavior change researchers were split into 4 groups of 4 or 5 people and for each session were provided with the descriptions of 2 or 3 BCTs per group. HCPs were asked to generate messages to address specific beliefs and concerns. The 31 BCTs and 7 beliefs and concerns presented in study 1 were those identified by rapid review [18] and considered to be plausible for delivery via brief messages. Three of the included beliefs and concerns presented to HCPs represented an additional 2 BCTs. “Difficulties remembering and understanding the medication regimen” represented the BCT 4.1: Instruction on how to perform a behavior and “perceived risks of taking medication” and “beliefs about medication necessity” represented BCT 5.1: Information about health consequences (Table 1). Each group had a moderator who did not actively participate in writing messages but asked participants to develop brief messages based on each BCT that would target tablet medication adherence for people with diabetes. The moderator then entered any messages that were agreed by the group onto a spreadsheet. The messages generated by behavior change researchers were reviewed by a different group of behavior change researchers and rated according to how relevant the BCT was thought to be to improving diabetes tablet medication adherence (on a scale of 1 to 10), how far the aim of getting 8 to 10 messages that reflect the BCT well had been achieved (scale of 1 to 10), and the fidelity of each message generated to the intended BCT (scale of 1 to 10). The group of HCPs remained as a single group throughout and rated their own messages for how well they thought the messages addressed the beliefs and concerns raised in the rapid review, how relevant the belief/concern was thought to be, and how well the aim of getting 8 to 10 messages that reflect the belief/concern well was reached (scale of 1 to 10).

In the fourth session, the ratings for how well each BCT or belief and concern met the aim of generating 8 to 10 good quality messages were ranked, and those that scored lowest were discussed with all participants as a single group. Suitability of the BCTs for delivery through text message was discussed. Participants were asked to complete a survey describing their level of expertise and a brief workshop evaluation form. Participants were given an honorarium for their time, and accommodation, travel, and other subsistence costs were reimbursed.

Following the workshop, only those BCTs that were deemed compatible with delivery by text message and suitable for medication adherence for people with type 2 diabetes with an appropriate score for fidelity to the BCT were retained and used in study 2.

### Study 2: Focus Group Study

#### Aim

The aim of the research was to ascertain the acceptability of the concept and types of messages to people with type 2 diabetes.

#### Recruitment

Five general practices sent letters introducing the study to eligible patients. Participants were eligible if they were aged over 18 years, taking tablet medication for type 2 diabetes, and had access to a mobile phone. Those who had been hospitalized in the last 3 months for hypo- or hyperglycemia, were pregnant or within 3 months postpartum, or had been diagnosed with a terminal illness were excluded.

#### Procedure

Participants were invited to take part in a focus group at the university or in a community location. Researchers facilitated the focus groups and presented the 31 BCTs that remained following study 1. As it would not be possible to discuss all 31 BCTs, these were grouped as potential strategies for behavior change grouped according to v1 taxonomy categories [15] with sample messages. As an example, BCT 1.2: Problem solving and BCT 1.4: Action planning are both grouped under goals and planning in the BCT v1 taxonomy [15]. Participants were given a description of the group “Messages that support and encourage you to plan to take your medication as intended, along with messages that encourage you to solve problems to help you achieve those goals” and some sample messages from those generated during study 1 were given (eg, “Plan when, where, and how you are going to take your medication” [BCT 1.4]).

The task of the groups was to discuss wording and acceptability of the idea of sending brief messages to encourage and support medication adherence overall rather than determine acceptability of individual messages or BCTs (the examples were given to provide a concrete grounding for discussion). Focus groups were recorded, transcribed, and analyzed thematically. Following the focus group study, suggestions made by participants were reviewed and acted on as appropriate.

### Study 3: Message Acceptability Survey

#### Aim

The goal of the research was to assess the acceptability of the BCTs and messages developed during studies 1 and 2 to people with type 2 diabetes.

#### Recruitment

Participants were aged over 18 years with type 2 diabetes and were taking tablet medication to control their diabetes (with or without concomitant insulin). Potential participants were invited from a nationwide database of individuals facilitated by the Greater Manchester Clinical Research Network. They had been diagnosed with diabetes, were interested in taking part in research, and had indicated on signing up to the database that they would be interested in completing online questionnaires (n=861). Advertisements were also posted on the Diabetes UK online support forum, and information about the study was distributed at a diabetes support group.

#### Procedure

The database facilitators sent initial email invitations containing a link to the participant information sheet, an online consent form, and 1 of 4 versions of the survey (see survey development and content section). Participants were screened for eligibility and required to agree with all consent statements before they could proceed to the survey. The advertisement also contained contact details if potential participants preferred to receive a paper version of the survey. In this case, the information sheet, consent forms, and 1 of the 4 surveys were printed and mailed to the individual with two postage paid return envelopes to keep consent forms and research data separate. No incentive was offered for completion.

#### Survey Development and Content

Two sets of messages from those developed in studies 1 and 2 were assessed for acceptability; 47 in survey 1, and 46 in survey 2. Messages were not grouped or ordered by BCT or belief/concern; both surveys were presented with messages in one order, and the reversed order, resulting in 4 versions of the survey. Participants were allocated sequentially to one of the versions of the survey. In total, 93 messages across the 31 BCTs and beliefs/concerns that remained after studies 1 and 2 were assessed (3 messages per BCT and belief/concern). Participants were asked to provide ratings for individual messages, and acceptability of each BCT or belief/concern was then considered across the 3 messages. The individual messages were selected by ordering per BCT or belief/concern according to the mean fidelity to the BCT or belief/concern score given to each message during study 1. For each BCT or belief/concern, one message that scored high, one that scored low, and one that was scored in the middle were chosen. This allowed examination of the acceptability across the range of fidelity to each BCT or belief/concern. Participants were asked initial screening questions, demographic questions, and whether they used a phone or tablet computer. Participants were then presented with either the 46 or 47 messages and after each message asked to provide 3 ratings of acceptability on 5-point Likert scales: How easy is the message to understand (very difficult to understand, difficult to understand, neither easy nor difficult to understand, easy to understand, very easy to understand)? How much do you like the message (do not like at all, do not like, neither like nor do not like, like, like a lot)? How useful would this message be for you (not useful at all to me, not useful to me, neither useful nor not useful to me, useful to me, very useful to me)? The final question was an open-ended comment box for any general comments about the messages. A panel of PPI representatives reviewed the survey, and changes were made in line with their suggestions prior to the survey being sent out. PPI representatives were reimbursed in accordance with the Involve guidelines from the National Institute of Health Research [19].

#### Analysis

For each facet of acceptability, individual message scores were examined to identify any messages that should be removed from the library. Mean scores for ease of understanding, liking, and usefulness were calculated across the messages for each BCT or belief/concern. An overall acceptability score was calculated as an average of the understanding, liking, and usefulness scores for each BCT or belief/concern.

Following the survey, only messages and BCTs, beliefs, and concerns that scored adequately in terms of acceptability were retained for study 4.

### Study 4: Message Fidelity Survey

#### Aim

The goal of the research was to assess the extent to which the messages with any amendments following studies 2 and 3 had fidelity to the intended BCTs.

#### Recruitment

Participants were expert researchers in behavior change. Potential participants were identified from those who were invited but unable to attend study 1 and the research team’s knowledge of those researching BCTs. Initial email invitations (n=32) were sent, and a single reminder email was sent to those who did not respond.

#### Procedure

If an individual responded positively to the invitation, they were sent a further email with a unique participant number, a link to the full participant information sheet, consent form, and 1 of 2 versions of the survey selected at random (one with the order of BCTs reversed). A random allocation sequence was generated in blocks of 10 using QuickCalcs (GraphPad Software), and the unique participant number was assigned the next allocation in the sequence. Participants had to agree to all consent statements before continuing to the survey. Participants were given an honorarium for completing the survey.

#### Survey Development and Content

Participants completed some brief questions about their expertise. For each BCT (n=26), participants were presented with the title and description of the BCT from the BCT v1 taxonomy [15], and then 6 sample messages were presented with “How well does this message reflect the BCT *problem solving* as defined above?” under each message (italics adjusted for each BCT). Answers were given on a 10-point scale anchored with 1 (not very well) to 10 (very well). As participants were behavior change researchers, ratings were not sought for the beliefs and concerns but rather on whether or not the message reflected an underlying BCT. Three of the beliefs and concerns represented an additional 2 BCTs, resulting in 26 BCTs (eg, messages developed for the concern “perceived risks of taking medication” were presented under BCT 5.1: Information about health consequences). See Table 1 for details of beliefs and concerns and associated BCTs. For each of the 26 BCTs, 6 messages were rated, totaling 156 messages. The 6 messages chosen were those rated as having the highest fidelity to the BCT during study 1. The final question was an open-ended comment box for any general comments about the messages.

## Results

### Study 1: Message Development Workshop

#### Participants

A total of 21 participants attended the workshop. Participants were employed as either researchers (13/21, 62%), both researchers and HCPs (7/21, 33%), or as HCPs (1/21, 5%). Participants had been paid to conduct research and/or health care professional work for between 6 and 40 years (mean 15.95 [SD 9.94]). All participants (n=21) described behavior change interventions as either central or somewhat central to their work, 62% (13/21) described medication adherence as central or somewhat central, and 43% (9/20; 1 missing) of participants described diabetes as central or somewhat central to their work. Participants had published between 2 and 50 papers (mean 14.82 [SD 13.47]) related to medication adherence, diabetes or behavior change interventions (19/21, 1 missing and 1 clinician marked N/A).

#### Behavior Change Techniques

A total of 371 messages were generated during the workshop; 356 were scored and 15 were generated as additional suggested messages by the participants when they had finished scoring. There were between 6 and 15 messages per BCT or belief/concern (mean 9.76 [SD 2.07]). All subsequent BCT codes reference the v1 BCT taxonomy [15]. On a scale of 1 to 10, perceived relevance of the BCT or belief/concern to tablet medication adherence for people with type 2 diabetes ranged from 4.00 (BCT 13.1: Identification of self as a role model) to 9.75 (BCT 8.3: Habit formation) with a median of 8.00. When asked the extent to which the aim of writing 8 to 10 messages that reflect the BCT or belief/concern well had been achieved (from 1=not at all achieved to 10=completely achieved), the mean scores across reviewers ranged from 3.25 (BCT 8.4: Habit reversal) to 9.25 (BCT 5.3: Information about social and environmental consequences) with a median of 7.00. Across the messages, each BCT or belief/concern received mean fidelity scores ranging from 3.50 (BCT 8.4: Habit reversal) to 8.58 (BCT 3.1 Social support [unspecified]) with a median of 8.00. See Table 1 for details.

#### Decisions for Message Development

Following discussions in the fourth session of the workshop, 7 BCTs were identified as either incompatible with delivery via brief message during the workshop or unsuitable for medication adherence for people with type 2 diabetes: BCT 8.4: Habit reversal; BCT 11.3: Conserving mental resources; BCT 12.2: Restructuring the social environment; BCT 13.1: Identification of self as a role model; BCT 13.2 Framing/reframing; BCT 13.3: Incompatible beliefs; and BCT 15.3: Focus on past success. For detailed reasons, see Table 2. Following study 1, the library contained 306 messages based on 24 BCTs and 7 beliefs and concerns (3 of these beliefs and concerns were based on an additional 2 BCTs).

### Study 2: Focus Group Study

#### Participants

A total of 23 participants with a mean age of 68 (SD 7.25) years and a mean of 11 (SD 5.94) years since diagnosis with type 2 diabetes took part in 1 of 5 focus groups; 17% (3/23) of the participants were female and 83% (15/23) were white British.

#### Behavior Change Techniques

Participants found the concept of sending messages related to medication adherence for diabetes acceptable, provided the messages were novel (eg, not using strategies the individual was already using). Participants were keen to introduce messages related to aspects of diabetes self-management beyond medication adherence and had suggestions for changes to the wording of some messages. The wider findings from these focus groups relating to the acceptability to people with type 2 diabetes of receiving short message service (SMS) messages in principle and what features the system should have are reported elsewhere [20].

#### Decisions for Message Development

Following the focus groups, suggestions were identified from the transcripts, and each was considered by the research team according to criteria adapted from previous research [21]. The criteria used to aid discussion were (1) how relevant the suggestion is to the target behavior, (2) how available the suggested content already is (if already widely available perhaps not needed in present intervention), (3) the ease of implementation from a technical perspective, and (4) support from theoretical findings and/or evidence. One researcher applied the criteria (with + for support, – for no support, and ? if unsure for each criteria on each suggestion); this was then brought to a multidisciplinary team meeting, agreements and disagreements about the rating were discussed, and a final decision was taken to accept or reject each suggestion. Following these discussions, messages related to other aspects of diabetes self-management were added in preparation for piloting (eg, links to verified sources of diet and exercise advice), and messages were reworded to use the word tablets rather than meds, medication, or pills in accordance with participants’ preference. The number of messages targeting medication adherence remained at 306, based on 24 BCTs and 7 beliefs and concerns (3 of these beliefs and concerns were based on an additional 2 BCTs).

**Table 1:** Number of messages, mean perceived relevance of the behavior change techniques (BCTs) to medication adherence, mean score for how well the aim of 8 to 10 messages that reflected the BCT well was met, and mean fidelity of messages to the intended BCT or belief/concern from study 1. BCTs are identified through the codes from BCT v1 taxonomy [15] and beliefs and concerns are labeled A through G.

BCT^a^ or belief/concern	Number of messages	Relevance to improving medication adherence for people with type 2 diabetes, mean (SD)	Aim to have 8 to 10 messages that reflect the BCT or belief/concern well, mean (SD)	Fidelity of messages to the intended BCT/belief or concern^b^, mean (SD)
1.2. Problem solving	10	8.00 (1.41)	7.00 (0)	7.18 (1.36)
1.4. Action planning	8	8.50 (0.71)	7.25 (0.96)	7.94 (1.92)
2.3. Self-monitoring of behavior	11	9.00 (1.55)	6.75 (0.96)	6.86 (1.94)
3.1. Social support (unspecified)	9	9.25 (0.96)	9.00 (0)	8.58 (1.40)
3.2. Social support (practical)	8	9.00 (0.82)	8.50 (1.29)	7.83 (1.42)
3.3. Social support (emotional)	9	7.67 (1.15)	7.50 (1.00)	7.72 (1.75)
4.2. Information about antecedents	6	8.50 (1.29)	8.75 (0.96)	8.17 (1.34)
5.3. Information about social and environmental consequences	13	7.50 (1.91)	9.25 (0.50)	8.50 (1.13)
5.5. Anticipated regret	11	7.50 (2.38)	7.50 (0.58)	7.34 (1.36)
5.6. Information about emotional consequences	7	7.67 (1.15)	7.75 (0.50)	7.36 (1.91)
6.2. Social comparison	11	6.00 (2.45)	7.75 (0.96)	6.85 (2.63)
6.3. Information about others’ approval	8	5.50 (3.54)	7.75 (1.71)	8.09 (1.84)
7.1. Prompts/cues	11	8.25 (0.96)	8.25 (0.50)	8.02 (1.34)
8.3. Habit formation	11	9.75 (0.50)	8.50 (1.00)	7.81 (1.48)
8.4. Habit reversal	3	4.50 (1.73)	3.25 (0.50)	3.50 (0.90)
9.1. Credible source	8	7.25 (2.22)	7.75 (0.50)	8.06 (1.64)
9.2. Pros and cons	11	6.67 (0.58)	8.25 (0.96)	8.31 (1.15)
9.3. Comparative imaginings of future outcomes	8	8.00 (1.00)	6.75 (0.50)	6.61 (1.99)
10.5. Social reward	12	7.50 (0.58)	8.50 (0.58)	7.71 (1.35)
11.2. Reduce negative emotions	8	6.50 (1.29)	6.75 (0.50)	6.97 (1.11)
11.3. Conserving mental resources	12	6.00 (2.16)	5.75 (1.26)	6.33 (1.02)
12.1. Restructuring the physical environment	13	9.00 (0.82)	8.50 (1.29)	8.25 (1.57)
12.2. Restructuring the social environment	9	6.00 (2.16)	4.75 (1.89)	7.00 (1.78)
13.1. Identification of self as role model	9	4.00 (2.45)	8.00 (0.82)	7.81 (2.14)
13.2. Framing/reframing	8	5.00 (1.00)	6.75 (0.50)	7.11 (2.16)
13.3. Incompatible beliefs	9	4.50 (3.87)	6.25 (1.71)	6.47 (1.92)
13.5. Identity associated with changed behavior	12	4.50 (3.00)	6.75 (0.50)	6.94 (2.44)
15.1. Verbal persuasion about capability	9	5.25 (0.50)	7.00 (0.82)	6.42 (2.06)
15.2. Mental rehearsal of successful performance	11	5.25 (0.96)	6.00 (0.82)	4.50 (2.82)
15.3. Focus on past success	8	6.75 (1.26)	7.50 (0.58)	7.00 (1.38)
15.4. Self-talk	5	5.25 (1.71)	8.50 (0.58)	7.70 (0.86)
A. Difficulties with side effects	8	8.25 (0.96)	7.75 (0.96)	7.06 (1.81)
B. Difficulties remembering and understanding the medication regimen (BCT 4.1: Instruction on how to perform a behavior)	8	7.50 (1.00)	7.50 (1.00)	7.22 (1.50)
C. Beliefs around medication in general and western medicines specifically	6	7.25 (0.50)	6.25 (0.96)	7.54 (1.28)
D. Perceived risks of taking medication (As E, BCT 5.1: Information about health consequences)	10	8.40 (0.89)	7.40 (0.55)	7.76 (1.02)
E. Beliefs about medication necessity (As D, BCT 5.1: Information about health consequences)	11	8.80 (1.30)	8.00 (0.71)	7.96 (1.07)
F. Social influence around taking medications	10	7.80 (0.84)	8.20 (0.45)	7.64 (1.24)
G. Health care system-related concerns	15	8.00 (0.71)	8.60 (0.89)	8.00 (1.01)

**^a^**BCT: behavior change technique.

**^b^**Mean is across reviewers per message and across messages per BCT.

**Table 2:** Reasons for exclusion of behavior change techniques following study 1.

Behavior change technique	Reason for exclusion
8.4. Habit reversal	Missing taking medication was not thought to be habit; therefore it was thought there was no habit to reverse in the sense psychologists think of habits
11.3. Conserving mental resources	Participants couldn’t find a way to operationalize this that wasn’t also habit formation. In addition, there was concern that suggesting people with type 2 diabetes focus on medication adherence alone could devalue other important lifestyle messages (eg, diet and physical activity) that are also key to diabetes self-management
12.2. Restructuring the social environment	Participants found it hard to structure messages that didn’t seem to suggest stopping seeing people
13.1. Identification of self as a role model	Participants did not feel a single health behavior such as taking medication as prescribed constituted an identity and therefore would be hard to model oneself as a role model
13.2. Framing/reframing	Hard to use without interaction; would need to know how someone is framing to start with in order to initiate change. This was not thought to be possible in a text message
13.3. Incompatible beliefs	Thought to be better used in a therapeutic situation rather than via text message
15.3. Focus on past success	Designed to react to what someone has said, this would be difficult to deliver outside of a therapeutic situation as the text message system would not know if someone had had past successes or not

### Study 3: Message Acceptability Survey

#### Participants

A total of 72 patients consented, of whom 11 rated no messages and 52 completed ratings for all messages. Patients who consented were aged between 30 and 83 (mean 62.0 [SD 11.3]) years, 39% (28/71; 1 participant did not specify) were female; 54% (39/72) of participants had had their last change in diabetes tablets over a year ago. See Table 3 for details and comparison with those who completed all the message reviews.

**Table 3:** Demographics and questionnaire responses for study 3 participants.

Characteristics		Consented (n=72)	Completed the message reviews (n=52)^a^
Female, n (%)^b^		28 (39)	21 (40)
Age in years, mean (SD)		61.99 (11.25)	62.81 (10.83)
**Ethnicity, n (%)**			
	White	64 (89)	45 (87)
	Asian/Asian British	1 (1)	0 (0)
	Mixed/multiple ethnic groups	1 (1)	1 (2)
	Did not answer	6 (8)	6 (12)
Time since diagnosis with diabetes in years, mean (SD)^c^		12.1 (5.38)	12.73 (5.76)
**Time since last change to diabetes tablets, n (%)^d^**			
	≤1 month	6 (8)	3 (6)
	>1 month to <3 months	6 (8)	6 (12)
	≥3 months to <6 months	8 (11)	8 (15)
	≥6 months to <1 year	9 (13)	6 (12)
	>1 year	39 (54)	27 (52)
	First prescription	3 (4)	1 (2)
	Did not answer	1 (1)	1 (2)

^a^Includes postal response and 1 respondent who answered questions about all but the last message.

^b^n=71 consented, n=51 completed due to missing value.

^c^n=38 consented, n=26 completed; there was a problem with one of the 4 questionnaires, this question did not allow respondents to answer, some answered in the next question and these are included here.

^d^n=71 consented, n=52 completed due to missing value.

#### Behavior Change Techniques

Mean scores for BCTs were highest for ease of understanding (median 4.24, range 3.71 to 4.60), then liking (median 3.33, range 2.92 to 3.85), and lowest for perceived usefulness (median 2.88, range 2.49 to 3.41). Overall acceptability (median 3.49, range 3.10 to 3.89) was over the midpoint of the scale (see Table 4).

#### Decisions for Message Development

As none of the BCTs scored below the midpoint on the scale (midpoint=3) for acceptability, it was decided to retain all the BCTs in the library. The number of messages in the library therefore remained at 306, based on 24 BCTs and 7 beliefs and concerns (3 of these beliefs and concerns were based on an additional 2 BCTs).

**Table 4:** Ease of understanding, liking, usefulness, and acceptability scores per behavior change technique or belief or concern from study 3. All available message reviews from participants (n=61) were analysed.

BCT^a^/belief or concern	Ease of understanding, mean (SD)	Liking, mean (SD)	Usefulness, mean (SD)	Overall acceptability, mean (SD)
1.2. Problem solving	4.13 (0.16)	3.33 (0.26)	2.84 (0.17)	3.43 (0.17)
1.4. Action planning	4.24 (0.09)	3.34 (0.18)	2.99 (0.20)	3.52 (0.12)
2.3. Self-monitoring of behavior	4.48 (0.06)	3.45 (0.25)	2.76 (0.20)	3.56 (0.13)
3.1. Social support (unspecified)	4.30 (0.17)	3.41 (0.06)	2.95 (0.17)	3.55 (0.11)
3.2. Social support (practical)	4.25 (0.16)	3.20 (0.08)	2.68 (0.28)	3.38 (0.05)
3.3. Social support (emotional)	4.22 (0.15)	3.27 (0.05)	2.91 (0.24)	3.47 (0.04)
4.2. Information about antecedents	4.43 (0.10)	3.68 (0.22)	3.25 (0.14)	3.79 (0.14)
5.3. Information about social and environmental consequences	4.38 (0.25)	3.28 (0.17)	2.85 (0.27)	3.50 (0.15)
5.5. Anticipated regret	4.46 (0.22)	2.95 (0.20)	2.88 (0.70)	3.43 (0.37)
5.6. Information about emotional consequences	4.08 (0.14)	3.35 (0.21)	3.04 (0.45)	3.49 (0.26)
6.2. Social comparison	4.18 (0.13)	3.25 (0.38)	2.79 (0.15)	3.41 (0.22)
6.3. Information about others’ approval	4.29 (0.15)	2.96 (0.43)	2.60 (0.42)	3.28 (0.24)
7.1. Prompts/cues	4.48 (0.10)	3.41 (0.26)	2.88 (0.30)	3.59 (0.18)
8.3. Habit formation	4.26 (0.21)	3.65 (0.10)	3.29 (0.40)	3.73 (0.13)
9.1. Credible source	4.53 (0.16)	3.56 (0.22)	3.11 (0.33)	3.73 (0.16)
9.2. Pros and cons	3.71 (0.56)	2.92 (0.42)	2.67 (0.31)	3.10 (0.41)
9.3. Comparative imaginings of future outcomes	3.92 (0.27)	3.12 (0.21)	2.93 (0.10)	3.32 (0.17)
10.5. Social reward	4.22 (0.53)	3.02 (0.39)	2.57 (0.43)	3.27 (0.45)
11.2. Reduce negative emotions	4.18 (0.06)	3.09 (0.27)	2.82 (0.14)	3.36 (0.10)
12.1. Restructuring the physical environment	4.60 (0.08)	3.62 (0.29)	3.09 (0.28)	3.77 (0.18)
13.5. Identity associated with changed behavior	4.00 (0.06)	3.20 (0.09)	2.93 (0.15)	3.38 (0.06)
15.1. Verbal persuasion about capability	4.22 (0.17)	3.32 (0.05)	2.73 (0.06)	3.43 (0.07)
15.2. Mental rehearsal of successful performance	3.87 (0.19)	2.95 (0.25)	2.57 (0.21)	3.13 (0.19)
15.4. Self-talk	4.35 (0.20)	3.25 (0.27)	2.88 (0.39)	3.49 (0.17)
A: Difficulties with side effects	4.40 (0.17)	3.85 (0.25)	3.41 (0.12)	3.89 (0.18)
B: Difficulties remembering and understanding the medication regimen (BCT 4.1: Instruction on how to perform a behavior)	4.11 (0.22)	3.33 (0.08)	2.83 (0.11)	3.42 (0.02)
C: Beliefs around medication in general and western medicines specifically	4.23 (0.10)	3.38 (0.16)	2.86 (0.06)	3.49 (0.09)
D: Perceived risks of taking medication (As E, BCT 5.1: Information about health consequences)	4.09 (0.56)	3.35 (0.55)	3.07 (0.45)	3.50 (0.52)
E: Beliefs about medication necessity (As D, BCT 5.1: Information about health consequences)	4.46 (0.12)	3.70 (0.05)	3.27 (0.09)	3.81 (0.03)
F: Social influence around taking medications (BCT 3.1: Social support unspecified)	4.35 (0.15)	3.23 (0.18)	2.49 (0.30)	3.36 (0.16)
G: Health care system–related concerns	4.04 (0.36)	3.48 (0.54)	3.10 (0.58)	3.54 (0.49)

^a^BCT: behavior change technique.

### Study 4: Message Fidelity Survey

#### Participants

A total of 18 participants completed the survey; all identified themselves as researchers (rather than clinicians or researchers and clinicians). They had been paid to do research for between 6 and 31 (mean 14.6 [SD 6.0]) years and had published between 5 and 110 papers in the areas of diabetes, medication adherence, or behavioral interventions (mean 29.2 [SD 29.0]). Behavior change interventions were rated as being central or somewhat central to all 18 participants. Medication adherence was also reported as central or somewhat central to 13 participants, and diabetes as central or somewhat central to 7 participants.

#### Behavior Change Techniques

The mean scores of fidelity to the intended BCT ranged from 5.53 (BCT 4.1: Instruction on how to perform a behavior) to 8.87 (BCT: 5.3 Information about social and environmental consequences) with a mean of 7.61 (SD 0.93). See Table 5.

#### Decisions for Message Development

As none of the BCTs fell below the midpoint on the scale for fidelity (midpoint=5.5) it was decided that all the BCTs examined could be adequately represented by brief messages. At this stage of development, the findings related to individual messages from studies 2, 3, and 4 were considered together. One message was removed as it had scored below the midpoint for acceptability to patients in study 3 and for fidelity to the intended BCT in study 4 (“Diabetes tablets do not wear off over time” intended to represent BCT 5.1: Information about health consequences).

The final stage of message development was to review each message from the library with HCPs working on the project to ensure there was nothing either misleading or that could contradict current medical advice. One BCT (10.5: Social reward) was removed as the messages relied on a link to pharmacy pick-up that was not possible from the message delivery system at this time. A further 3 messages were removed and 7 were amended as a result of this review (see Table 6 for reasons). The final library contained 290 messages based on 25 BCTs and 4 beliefs and concerns (see Table B in Multimedia Appendix 1 for sample messages and Figure 1 for a flowchart of the message development process).

**Table 5:** Fidelity of the messages to the intended behavior change techniques from study 4.

Behavior change technique	Fidelity of messages mean (SD)
1.2. Problem solving	8.73 (0.54)
1.4. Action planning	7.54 (2.22)
2.3. Self-monitoring of behavior	7.79 (0.80)
3.1. Social support (unspecified)	7.91 (0.74)
3.2. Social support (practical)	8.73 (0.41)
3.3. Social support (emotional)	8.07 (0.20)
4.1. Instruction on how to perform a behavior	5.53 (0.93)
4.2. Information about antecedents	6.43 (0.62)
5.1. Information about health consequences	6.49 (1.97)
5.3. Information about social and environmental consequences	8.87 (0.19)
5.5. Anticipated regret	8.43 (1.16)
5.6. Information about emotional consequences	6.67 (1.69)
6.2. Social comparison	7.17 (1.45)
6.3. Information about others’ approval	8.44 (0.92)
7.1. Prompts/cues	8.32 (0.45)
8.3. Habit formation	8.32 (1.06)
9.1. Credible source	7.49 (1.71)
9.2. Pros and cons	8.46 (0.65)
9.3. Comparative imaginings of future outcomes	7.02 (1.81)
10.5. Social reward	5.76 (0.55)
11.2. Reduce negative emotions	6.73 (0.49)
12.1. Restructuring the physical environment	8.40 (0.51)
13.5. Identity associated with changed behavior	7.03 (1.18)
15.1. Verbal persuasion about capability	7.75 (1.64)
15.2. Mental rehearsal of successful performance	7.48 (0.97)
15.4. Self-talk	8.13 (0.88)

**Table 6:** Reasons for message removal or amendment following health care provider review.

Message	BCT^a^	Action	Reason
If your blood sugar gets out of control, it could put you on an emotional rollercoaster. Taking your tablets as often as you should will stop the ups and downs.	5.6. Information about emotional consequences	Removed	Message implies a direct link between blood sugar control and an emotional rollercoaster—this is not true
Some people with diabetes who don't take their tablets lose their foot. Imagine how you feel if you let this happen to yourself.	5.5. Anticipated regret	Removed	Message implies a direct link between not taking tablets and losing a foot—smoking is a bigger risk factor.
Most religious leaders would agree that taking diabetes medication regularly is important.	9.1. Credible sources	Removed	Message flagged in qualitative comments of study 3; was not thought to be wholly accurate.
The tablets prescribed for your diabetes are very effective and can work alongside other treatments. Please let us know of anything else you may be taking.	C. Beliefs around medication in general and western medicines specifically	Amended	Confusing whom the message is seen as coming from.
If you don’t understand don’t be afraid to say so. There’s a team of people here to help you.	G. Health care system-related concerns	Amended	Confusing whom the message is seen as coming from.
Taking your tablets can be as routine as having your morning coffee. Use this time as a prompt, and make your tablet taking a habit.	8.3. Habit formation	Amended	May have a morning coffee at 11:00 without any food—tablets should be taken with food.
Don’t end up feeling guilty about extra diabetes complications caused by not taking your tablets as prescribed.	11.2. Reduce negative emotions	Amended	Complications may not be caused by not taking your tablets; referring to increased risk of complications is more accurate
If you lost your eyesight because you didn't take your diabetic tablets, would you regret it?	5.5. Anticipated regret	Amended	Loss is very final, seen to be too negative as this may go to people who have eyesight problems and these may not be linked to diabetic medication—a link implied that isn’t necessarily there.
If you take your tablets as prescribed, it reduces your risk of serious complications by 50% (Diabetes UK).	9.1. Credible sources	Amended	Quantification hard to substantiate, so removed
Diabetes UK: Forgetting diabetes tablets just twice a week halves their overall benefit (hyperlink to webpage).	9.1. Credible sources	Amended	Quantification hard to substantiate, so removed

## Discussion

Following this systematic development process involving four studies, we conclude that the messages produced have good acceptability and fidelity to their intended BCT. The retained BCTs were shown to be appropriate for delivery through brief messages, acceptable to patients, and clearly understood. All mean scores for retained BCTs were above the median point on the scales in studies 1, 3, and 4.

There are at least three key strengths of this research. First and most important, the systematic transparent process of message development allows for clarity in terms of message content; this will aid interpretation of any intervention effects. Second, the inclusion of people with type 2 diabetes, experts in behavior change research, and health care practitioners to ensure views of key stakeholders are incorporated. Third, as a consequence of the first two strengths, this research has resulted in the identification of BCTs appropriate for delivery through brief messages and development of a library of messages with evidence supporting their acceptability and fidelity to explicit BCTs that can be used in further research.

The majority of the sample of people with type 2 diabetes recruited for studies 2 and 3 were of white ethnicity, and this is acknowledged as a limitation. However, the work reported here is part of a larger program of work to develop evidence-based brief messaging for type 2 diabetes. Due to the increased risk of the condition among South Asian populations [22], these populations are a specific focus of this work. Focus groups with South Asian populations were run in parallel with those described in study 2 [23]. Further development will be needed to explore the similarities and differences between these two focus group studies and the potential tailoring, or additional messages needed, to ensure a library of messages that are acceptable to the whole population of people with type 2 diabetes.

As identified in a Cochrane review, novel approaches to medication adherence are needed [4]. We were therefore keen at this stage to include as many potentially effective components as possible for further testing in a trial setting while excluding any messages or BCTs that were definitely unacceptable to the target population. Currently there is not a widely recognized threshold for acceptability for these studies. A threshold for acceptability was set at the median point or above [24], as this seemed appropriate to meet our aims. In a recent definition of acceptability, cognitive and emotional reactions to an intervention and perceived relevance were identified as key facets in the definition [25]. In addition to formative assessment of the acceptability of the broad concepts used in study 2, these individual facets have been measured in study 3 through ease of understanding, liking, and perceived usefulness to an individual. Of the facets of acceptability measured, perceived usefulness scored lower than either liking or ease of understanding. It should be noted that at this stage in development it was only possible to measure anticipated acceptability. As conceptualized by Sekhon and colleagues [25], acceptability should be measured across development of an intervention to assess both anticipated and experienced acceptability. It could be hypothesized that of the facets measured, perceived usefulness is the hardest to prospectively measure accurately. Future research could explore whether certain approaches may be perceived as more or less useful to different people in different contexts as this might be more appropriately done when people have had a chance to use the intervention in a more real-world setting and can report experienced acceptability. Future research could also explore the relationship between anticipated acceptability as we have measured here and experienced acceptability when participants are receiving these messages as part of their day-to-day lives.

Previous research in this area has found modest improvements in glycemic control in those with poorly controlled diabetes as a result of receiving brief diabetes-related messages in conjunction with blood glucose monitoring [26]. The messages in this previous research were generated within the research team and based on a review of previous research, existing mobile health interventions, and current patient resources [27]. The intervention was described as informed by 2 behavior change theories, and researchers incorporated 8 BCTs from an early version of the taxonomy [14] into the messages including “prompting self-monitoring” and “providing general encouragement” as well as reminders and feedback on blood glucose results through messages and an accompanying website [27]. It is not clear from the publications how the theories and BCTs were selected from the sources of information used (eg, the review, mobile health interventions, and patient resources). The messages were reviewed by diabetes experts and people with type 2 diabetes and piloted for acceptability and usability [27]. By contrast, the 4 studies reported in our research expand on this previous research by using a wider pool of individuals to generate the messages, looking beyond the individual theories and BCTs currently used to incorporate a wider range and back-checking with a separate group of researchers that the messages are good exemplars of the intended BCTs.

This approach may lead to more novelty in brief message interventions and greater confidence that individuals who receive the messages will be receiving the intended technique. This is important because if the intervention is found to be either effective or not effective, it will allow researchers to explain why, which will add to the empirical evidence in this area and allow researchers to optimize the intervention. Although some of the BCTs identified in the rapid review were deemed by behavior change researchers and health care professionals to be difficult to deliver through brief messages, we retained 29 BCTs and beliefs and concerns through to the final message library. This ensures that in addition to the more commonly used BCTs such as “self-monitoring of behavior,” this intervention can explore approaches to this problem that are novel in brief message interventions.

This research provides a transparent development process for other researchers to use or improve upon. While this approach is not intended to be an alternative to other approaches of overall intervention development, to the authors’ knowledge there is currently no guide for systematically developing the content of messages used in self-management interventions and ensuring that the content accurately represents the intended BCTs. This approach could therefore be used in conjunction with other approaches to intervention development [8]. The components of this approach were all important, however the order the studies were undertaken could have been altered. Initially, we planned to conduct the focus group study (reported here as study 2) prior to the message development study (reported here as study 1). As one of the aims of the focus group study was to ascertain people with diabetes’ views on a system such as this, if these had been negative, the time and effort in developing the messages may have been wasted. However, due to delays in recruitment for the focus group study, we decided to go ahead with the message development study for two reasons: (1) the brief messages developed could be delivered in a number of ways (if participants had favored an app or Web-based system or even a paper-based system, the text of the messages could still have been used) and (2) it is easier for people to give opinions on a proposed system if they are provided with concrete examples. The opinions given by participants in study 2 were more useful as they were of actual messages we were planning to use rather than any that had been generated within the team. Overall, the process was relatively time consuming compared with other ways of generating messages; however, this is considered justified for the confidence we can now have that these messages are acceptable and have fidelity to the intended BCTs. Furthermore, there is the potential that some of this work could be adapted to other medication adherence interventions without repeating all stages; this may be less time consuming than developing those interventions from scratch. There were some decisions the team made about BCTs to include based on the available technology, which is constantly evolving. Because we have transparently presented the BCTs considered and reasons for exclusion, we hope if other researchers are considering other forms of technology or systems with a greater number of components, the excluded BCTs could duly be reconsidered.

Through developing a library of brief messages that are based on explicit BCTs and have been shown to be acceptable to a patient population, this research could also provide a valuable resource to develop theory in the area of medication adherence and diabetes and to optimize brief message interventions for this population. In a recently published review study, researchers explored how BCTs might have an effect on behavior by extracting the proposed links between BCTs and mechanisms of action [28]. However, the authors noted that the links proposed by intervention study authors were rarely tested empirically. Due to the fidelity of the messages to explicit BCTs, the messages developed here could be used to test theories of behavior change and the relationships between BCTs, mechanisms of action, and behavior change. The message library developed here will be further refined through our forthcoming pilot and feasibility work. Although messages were found to have good fidelity to their intended BCT, it was raised in qualitative comments that a single message may represent more than one BCT from within the taxonomy [15]; this could be explored in future research. In addition, it was recognized that not all BCTs are suitable for delivery through brief messages. Specifically, those requiring interaction could not be facilitated by the current system. Further research could explore more advanced systems that could deliver these BCTs (eg, chat bots to allow lifelike automated responses during interactions), but it must be accepted that there may be some BCTs that are better delivered in person. In these cases, future research could explore the possibility of incorporating any of these techniques into medication reviews with an HCP.

Participants in the study 2 focus groups requested messages around diet and exercise management in addition to messages focused on medication adherence. A wariness was also expressed by the participants in study 1 that focusing solely on medication adherence may have the unintended effect of making other aspects of diabetes self-management seem less important. Messages that provide information from verified sources related to other aspects of diabetes care therefore may form an important part of engaging someone with the system and ensure a more holistic view of the condition. However, this will create a challenge when interpreting results and exploring the mechanisms of action. In addition, the focus here was on tablet medication adherence; further research would be needed to assess whether any of these messages would be acceptable and appropriate for people using only injected medication or what adaptations would be needed to ensure relevance. Our future work will aim to explore the potential mechanisms of action both qualitatively and quantitatively with participants while using the messaging system.

In conclusion, a library of brief messages acceptable to people with type 2 diabetes representing explicit BCTs has been developed using a rigorous, transparent process. This will provide the basis for a novel brief message intervention to improve medication adherence in people with type 2 diabetes and can be further used to develop the theory and understanding of behavior change in this area.

## Appendix

Multimedia Appendix 1 Supplementary tables.

## Abbreviations

BCT: behavior change technique
HCP: health care professional
NHS: National Health Service
NIHR: National Institute for Health Research
PPI: Patient and Public Involvement panel
SMS: short message service
