# Supplementary material for: Use of Brief Messages Based on Behavior Change Techniques to Encourage Medication Adherence in People With Type 2 Diabetes: Developmental Studies
Source: J Med Internet Res. 2020 May 13;22(5):e15989. doi: 10.2196/15989 (PMC7254292; doi:10.2196/15989)
Supplement: Multimedia Appendix 1 [file jmir_v22i5e15989_app1.docx]

Multimedia Appendix 1

Table A: BCTs that were excluded between the rapid review [18] and study 1 with reasons

| BCT | Reason for exclusion |
| --- | --- |
| 2.4. Self-monitoring of outcome(s) of behaviour | HbAc1 is a measure of blood glucose levels over a period of weeks/months and a measurable outcome of medication adherence. However, HbAc1 readings change slowly and patients may experience delayed appreciation and benefit of their adherent behaviour, which could be demotivating. Self-monitoring of blood glucose is an alternative outcome of adherence behaviour, but evidence is not convincing that self-monitoring blood glucose improves glycaemic control for people using oral medication (Farmer et al., 2007). |
| 2.7. Feedback on outcome(s) of behaviour | It is arguably not feasible to do this with basic SMS systems as it would require i) an inbuilt feature that enables patients to record adherence behaviour, i.e. HbAc1 or blood glucose readings, and ii) an inbuilt bi-directional messaging feature that enables patient-physician interaction to provide tailored feedback via SMS message. |
| 4.3. Re-attribution | A key element of this BCT is eliciting the patient’s perceptions around the causes of behaviour and to suggest alternative explanations; it is not considered feasible to elicit such perceptions and suggest alternatives through an SMS system. |
| 4.4. Behavioural experiments | Too much to do in the standard 160-character limit of an SMS message. |
| 5.2. Salience of consequences | The taxonomy states that this BCT must go beyond *5.1. Information about health consequences* in order to be discrete, but it is not clear how this would be possible in an SMS message format. Plausibly covered by *5.1. Information about health consequences.* |
| 5.4. Monitoring of emotional consequences | Following conversation with AF and RR, it appeared that people with T2DM only feel symptoms/ill when their blood sugars are low. Therefore, people can feel fine even when their blood sugar is not managed well (e.g. too high). The relationship between somatic symptoms and blood glucose does not always seem straightforward. Plausibly covered by *5.6. Information about emotional consequences* |
| 6.1. Demonstration of behaviour | It is not possible to provide an observable example of the performance of medication adherence behaviour via a 160-character SMS message. |
| 8.1. Behavioural practice/rehearsal | Medication taking is arguably not a complicated behaviour to practice, nor do we want to encourage medication taking in a context or at a time when the behaviour may not be necessary for risk of overmedication |
| 8.6. Generalisation of target behaviour | In the context of an SMS medium, this BCT is plausibly covered by and/or has overlaps with *8.3. Habit formation, 8.4. Habit reversal,* and *12.2. Restructuring the social environment.* |
| 8.7. Graded tasks | It is not clear what gradients the tasks could be staggered at; this may require a degree of tailoring to individual patients and this interactive feature may not be possible in an SMS system. |
| 12.3. Avoidance/ reducing exposure to cues for the behaviour | It will be difficult to establish what specific cues lead to avoidance/reduction in medication adherence without a conversation with the patient. The included qualitative reviews revealed that patients’ avoidance cues can often exist in social circles and at social events and thus we believe the pertinent aspects of this BCT will be covered by *12.2. Restructuring the social environment.* |
| 12.5. Adding objects to the environment | We are hesitant to ask patients to add objects they do not already own, i.e. ask them to buy a pill box to organise their medication. |
| 13.4. Valued self-identity | This BCT may be best reflected in 2+, linked SMS messages. Furthermore, it is not clear where the patient’s would acquire the rating scales needed to fulfil this BCT. |

Table B: Example messages with associated Behaviour Change Technique (BCT)

| Target and category of message | BCT/ Belief or concern | Example messages |
| --- | --- | --- |
| Medication adherence, BCT | 1.4 Action Planning | Plan when, where and how you are going to take your medication. |
| Medication adherence, BCT | 15.1 Verbal persuasion about capability | If you are struggling with your diabetes tablets then don't worry, you will be able to master it in time.  You will get on top of it. |
| Medication adherence, BCT | 7.1 Prompts/ cues | It can be difficult to remember to take your tablets. Why not set an alarm to remind you to take them? |
| Medication adherence, beliefs and concerns | G Healthcare system related concerns | Lots of questions? Check who the best person to see might be |
| Diet management | Signposting | Stuck for new ideas? You can search recipes for mains, desserts and snacks online at [Diabetes.org.uk](http://Diabetes.org.uk) |
